# CCL4 is the only predictor for non-responder in GT-1 CHC patients with favorable IL28B genotype when treated with PegIFN/RBV

**DOI:** 10.1186/s12876-017-0724-4

**Published:** 2017-12-29

**Authors:** Chia-Chen Lin, Shih-Huan Su, Wen-Juei Jeng, Chien-Hao Huang, Wei Teng, Wei-Ting Chen, Yi-Cheng Chen, Chun-Yen Lin, I-Shyan Sheen

**Affiliations:** 1 School of Medicine, College of Medicine, Chang-Gung University, 5, Fu-Xin street, Quain San, TaoYuan, 330 Taiwan; 2 Division of Hepatology, Department of HepatoGastroenterology, Chang-Gung Memorial Hospital, Linkou Medical Center, TaoYuan, Taiwan

**Keywords:** Chemokines, Cytokines, Treatment response, Chronic hepatitis C, Genotype-1, Interleukin-28B polymorphism

## Abstract

**Background:**

Chemokines/cytokines play important roles in the pathogenesis of chronic hepatitis C (CHC). However, their clinical characteristics and implications in treatment responses to pegylated interferon plus ribavirin treatment (PegIFN/RBV) have not been fully illustrated yet. In this study, we intended to investigate the possible predictability of serum chemokines/cytokines on the treatment response in Taiwanese of CHC, genotype-1 (GT-1).

**Methods:**

60 Patients with GT-1 CHC infection who had been treated with PegIFN/RBV were enrolled, including 27 (45%) with sustained virological response (SVR), 11 (18%) with relapse after 48 weeks of treatment and 22 (37%) non-response (NR). Clinical parameters, seven chemokines/cytokines, CCL3, CCL4, CXCL9, CXCL10, CXCL11, IL-10 and IFN-γ, and genotypes of rs12979860, the single nucleotide polymorphisms (SNPs) of interleukin-28B (IL28B) were analyzed for their relationship to treatment response.

**Results:**

Baseline serum levels of CXCL10, CXCL11, CCL3 and CCL4 were significantly higher in NR group while comparing with non-NR group. (CXCL10: *p* = 0.001; CXCL11: *p* < 0.001; CCL3: *p* = 0.006; CCL4: *p* = 0.005). However, only rs12979860 CC genotype was the independent factors for NR in GT-1 CHC infection (OR, 8.985; *p* = 0.008). In addition, baseline serum level of CCL4 was found to be the only independent factor for NR in GT-1 CHC patients with favorable IL28B genotype (OR, 1.134; *p* = 0.039).

**Conclusions:**

IL28B genotype is the predictor for NR in GT-1 CHC patients treated with PegIFN/RBV, while baseline serum level of CCL4 is the only predictor for NR in GT-1 CHC patients with favorable IL28B genotype.

## Background

Chronic hepatitis C is currently one of the leading causes of cirrhosis and hepatocellular carcinoma (HCC) in the whole-wide world [1, 2]. Eradication of HCV virus infection could reduce the risk of cirrhosis, hepatocellular carcinoma and hepatic decompensation [3, 4]. Though the direct antiviral agents (DAAs) are now the standard of care in Western countries [5], dual therapy of pegylated interferon-α/ribavirin (PegIFN/RBV) still is a popular and effective treatment in several countries where DAAs are not available or not affordable [6–8]. In the treatment with PegIFN/RBV, the patients with non-response (NR) are a troublesome group of patients [9]. Even in the era of DAAs, NR group also highlight a special group of patient that needs special attention [10]. Recently, in the present newer generation of DAAs, this group of patients has finally achieved satisfactory SVR rate. However, in the next development of chronic hepatitis C treatment, shorter duration of interferon-free DAAs will be a hot issue to be investigated [11]. In this possible new trend of treatment development, this potential NR group is worthy of re-revaluation.

Host immune response strongly correlates to the success of antiviral treatment. According to the previous studies, chemokines/cytokines do play important roles in the pathogenesis of chronic hepatitis C. Chemokines and chemokine receptors are crucial in T cell recruitment into infected sites and are involved in inflammation, infection and tissue damage [12, 13]. Type I interferons upregulate either directly or indirectly the expression of CCL3–5, which were potent ligands of the chemokine receptors CCR5 and CCR1. Similarly, Type II interferons are recognized as the most potent inducers of CXCL9–10, which bind to the chemokine receptor-CXCR3 [13]. A previous study revealed that the predominant liver infiltration by majorly CCR5 high/ CXCR3 high phenotype CD8+ lymphocytes in GT-1 CHC patients correlates to intrahepatic chemokine expression level and the inflammatory activity of chronic hepatitis C [14, 15]. However, the clinical implications in treatment responses to pegylated interferon plus ribavirin (PegIFN/RBV) treatment have not yet been fully illustrated. In the era of PegIFN/RBV treatment, the treatment would be terminated if HCV RNA still detectable by 24 weeks (so-called NR). The host immune reaction between non-responder and responder under Peg-IFN/RBV remained unclear. Here, we examined the impact of cytokine and chemokine (CXCL9, CXCL10, CXCL11, CCL3, CCL4, IFN-γ and IL10) from peripheral blood mononuclear cells between NR and non-NR to elucidate why host immune failed to response toward PegIFN/RBV treatment.

## Methods

### Patient recruitment

We retrospectively analyzed naive GT-1 CHC patients who had been treated with PegIFN/RBV at Chang Gung Memorial Hospital, Linkou Medical center with available stored serum between 2011 and 2013. There were 22 patients with treatment outcome of non-responder. Therefore, 38 age and gender matched non-NR patients with stored serum were recruited as well (Table 1). Patients with other concomitant liver diseases, such as hepatitis B virus, human immunodeficiency virus, alcoholic liver disease, and autoimmune hepatitis, were excluded. Liver cirrhosis was evaluated by liver biopsy or by FIB-4.

**Table 1:** Baseline Characteristics of CHC, GT1 Patients

Variables	Overall	NR (22)^a^	non-NR (38)^a^	*P* value
Age (years)	58.23 ± 9.19	55.6 ± 9.2	59.8 ± 9.0	0.090
Male (%)	55.0	50.0	57.9	0.599
BMI (Kg/m^2^)	25.44 ± 3.22	25.5 ± 3.7	25.4 ± 2.9	0.865
AST (U/L)	80.85 ± 40.27	91 ± 48	75 ± 34	0.118
ALT (U/L)	105.63 ± 59.86	108 ± 53	104 ± 64	0.797
HCV RNA (log_10_ IU/ml)	3.38(5.17)^b^	2.48(4.74)^b^	3.72(5.66)^b^	0.591
Diabetes Mellitus (%)	25.0	13.6	31.6	0.215
IL28B (CC %)	75.0	50.0	89.5	**0.001**
Liver cirrhosis (%)	28.3	45.5	18.4	**0.038**

^a^number of patients shown in parentheses

^b^median (IQR) shown in parentheses

Data are shown as mean ± standard deviation. Statistic analysis was done by Mann-Whitney test for comparison. Significant *P* values are shown in bold. AST, aspartate aminotransferase; ALT, alanine aminotransferase; HCV, hepatitis C virus; IL28B, Interleukin-28B

The treatment regimens of our patients were standard weight-based pegylated interferon plus ribavirin (PegIFN/RBV) treatment (peginterferon alfa-2a (180 mcg/week) or peginterferon alfa-2b (1.5 mcg/kg/week) subcutaneously plus weight-based ribavirin (1000 mg/d for weight < 75 kg and 1200 mg/d for weight > 75 kg)). Patients who did not fulfill the 80/80/80 adherence rule were excluded. Patients with no rapid virological response (RVR) had received a 48-week treatment while 24-week treatment for patients with RVR and low baseline viral load (HCV-RNA <0.4 × 106 IU/ml). No early virological responses (EVR) as the stop rule was applied to the treatment regimen. Treatment was terminated if detectable HCV-RNA at week 24 weeks.

Definitions of the treatment responses by serum level of HCV-RNA, assessed according to international definitions, were undetectable HCV-RNA 24 weeks after the cessation of treatment as sustained virological response (SVR), positive HCV-RNA at the end of at least 24 weeks of treatment as NR, and positive HCV-RNA after 48 weeks of treatment as relapser.

### Laboratory assay

The HCV-RNA levels were measured by commercial quantitative polymerase chain reaction (PCR) assay, either VERSANT HCV RNA 3.0. Assay (HCV 3.0 bDNA assay, Bayer Diagnostics, Berkeley, Calif., lower limit of detection: 5.2 × 102 IU/ml) or COBAS TaqMan HCV Test (TaqMan HCV; Roche Molecular Systems Inc., Branchburg, N.J., lower limit of detection: 15 IU/ml). Serum sample was tested further by COBAS® AMPLICOR HCV Test, v2.0 (CA V2.0, Roche Diagnostic Systems., lower limit of detection: 50 IU/ml) if non-detection of HCV-RNA by VERSANT HCV RNA 3.0. Assay. HCV genotype was determined by a genotype specific probe based assay in the 5′ untranslated region (LiPA; Innogenetics, Ghent, Belgium).

Seven chemokines and cytokines assessed in this study were CXCL9–11, CCL3–4, IL-10 and IFN-γ. Serum samples were analyzed by BD Cytometric Bead Array Human Inflammatory Cytokines Kit, produced by Becton, Dickinson and Company BD Biosciences, U.S.

### Genomic DNA extraction and IL28 B genotyping

Anti-coagulated peripheral blood was obtained from HCV patients. Genomic DNA was isolated from EDTA anti-coagulated peripheral blood using the Puregene DNA isolation kit (Gentra Systems, Minneapolis, MN) as previously described. The oligonucleotide sequences flanking ten IL28B polymorphisms were designed as primers for Taqman allelic discrimination. The allele specific primers for rs12979860 were labeled with a fluorescent dye (FAM and VIC) and used in the PCR reaction. Aliquots of the PCR product were genotyped with allele specific probe of SNPs using real-time PCR (ABI).

### Ethics statements

All patients in this study provided written informed consent. The study protocol conformed to the ethical guidelines of the 1975 Declaration of Helsinki and was approved by the ethical committees of Chang Gung Memorial Hospital.

### Statistical analysis

Chi-square test was used to compare the categorical variables of the groups. Continuous variables were compared with student’s t test or Mann-Whitney U test. Logistic regression analyses for predictors of treatment response were conducted using patients’ demographic, clinical variables, IL28B SNPs and serum levels of chemokines/cytokines. The clinical variables included gender, age, viral load of HCV-RNA, grading of modified HAI and fibrosis stages, body mass index (BMI), Glycohemoglobin (HbA1c), aspartate aminotransferase (AST), alanine aminotransferase (ALT), and rs12979860 SNPs. The odds ratios (OR) and 95% confidence intervals (95% CI) were also calculated. All *P* values less than 0.05 by the two-tailed test were considered statistically significant. Variables that achieved a statistical significance less than 0.10 on univariate analysis were entered into multivariate logistic regression analysis to identify the significant independent predictive factors. All statistical analyses were performed with statistical software, SPSS for Windows (version 19, SPSS. Inc., Chicago, IL, USA).

## Results

### Patients’ characteristics

A total of 60 patients with chronic hepatitis C genotype 1 infection were recruited into analysis. The majority of the patients are non-cirrhotic (71.7%) and more than half are male (55%). Twenty-two patients were NR and the other 38 are responders (non-NR) (including 27 patients with SVR and11 patients with relapse after 48 weeks of treatment (relapser) (Table 1).

By comparison of baseline characteristics, there were no significant differences between NR and non-NR groups in terms of age, gender, BMI, baseline viral load, serum levels of liver enzymes and diabetes mellitus. However, the frequency of IL28B-related rs12979860 CC genotype in NR group was significantly lower than that in non-NR group (NR vs. Non-NR: 50.0% vs. 89.5%, *p* = 0.001). In addition, a significantly higher percentage of liver cirrhosis was associated in non-response group (NR vs. non-NR = 45.5% vs. 18.4%, *p* = 0.038) (Table 1).

### IL28B genotype is the only predictor for NR

**Fig. 1:**
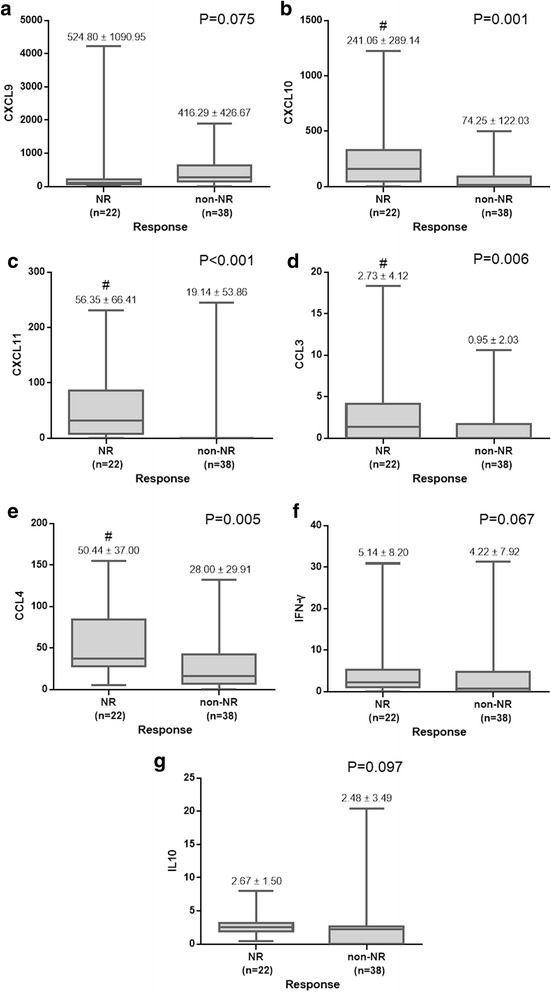
Comparison of Baseline Serum Levels of Chemokines/Cytokines of CHC, GT1 patients**. (a) (f) (g)** Pretreatment serum levels of CXCL9 (*P* = 0.075), IFN-γ (*P* = 0.067) and IL-10 (*P* = 0.097) showed no significant differences between NR and non-NR group of patients. **(b) (c) (d) (e)** NR group of patients showed higher baseline levels of CXCL10 (#*P* = 0.001), CXCL11 (#*P* < 0.001), CCL3 (#*P* = 0.006) and CCL4 (#*P* = 0.005) than non-NR group. Significance was assessed by means of the nonparametric Mann–Whitney test. Box plots represent medians and 25th–75th percentiles

The baseline pre-treatment level of chemokines/cytokines, including CXCL9, CXCL10, CXCL11, CCL3, CCL4, IFN-γ and IL10 were measured between NR and non-NR. CXCL10, CXCL11, CCL3 and CCL 4 were significantly higher in NR group while comparing with non-NR group (CXCL10: NR vs. non-NR = 241.06 ± 289.14 vs. 74.25 ± 122.03, p = 0.001; CXCL11: NR vs. non-NR = 56.35 ± 66.41 vs. 19.14 ± 53.86, *p* < 0.001; CCL3: NR vs. non-NR = 2.73 ± 4.12 vs. 0.95 ± 2.03, *p* = 0.006; CCL4: NR vs. non-NR = 50.44 ± 37.00 vs. 28.00 ± 29.91, *p* = 0.005) (figure 1).

Furthermore, the impacts of these chemokines/cytokines were evaluated along with baseline clinical factors by logistic regression analysis. By univariate logistic regression analysis (Table 2), rs12979860 CC genotype, CXCL10, CXCL11, CCL3, CCL4 and liver cirrhosis were the factors for non-NR. However, rs12979860 CC genotype was the only independent factor for NR by multivariate logistic analysis. (Table 2).

**Table 2:** Predictors of NR in the patients of CHC GT1, Treated with P/R by univariate and multivariate Logistic regression analysis

Variables	UV			MV		
	OR	95%C.I	*P* value	OR	95%C.I	*P* value
IL28B	8.500	2.246–32.174	**0.002**	8.985	1.778–45.406	**0.008**
CXCL9	1.000	0.999–1.001	0.584			
CXCL10	1.005	1.001–1.009	**0.012**	1.004	0.997–1.011	0.292
CXCL11	1.011	1.001–1.021	**0.039**	0.999	0.985–1.013	0.839
CCL3	1.270	0.987–1.634	**0.064**	1.292	0.910–1.835	0.152
CCL4	1.021	1.003–1.039	**0.022**	1.011	0.980–1.042	0.500
IFN-γ	1.015	1.022–0.696	0.663			
IL10	1.023	0.845–0.397	0.800			
Liver cirrhosis	0.271	0.084–0.876	**0.029**	0.267	0.058–1.223	0.089

*UV* Univariate logistic regression analysis. *MV* Multivariate logistic regression analysis. *OR* Odds ratio, *CI* Confidence interval

Significant *P* values are shown in bold

### CCL4 is the only predictor in CHC GT1 patients with advantageous IL28B genotype

**Fig. 2:**
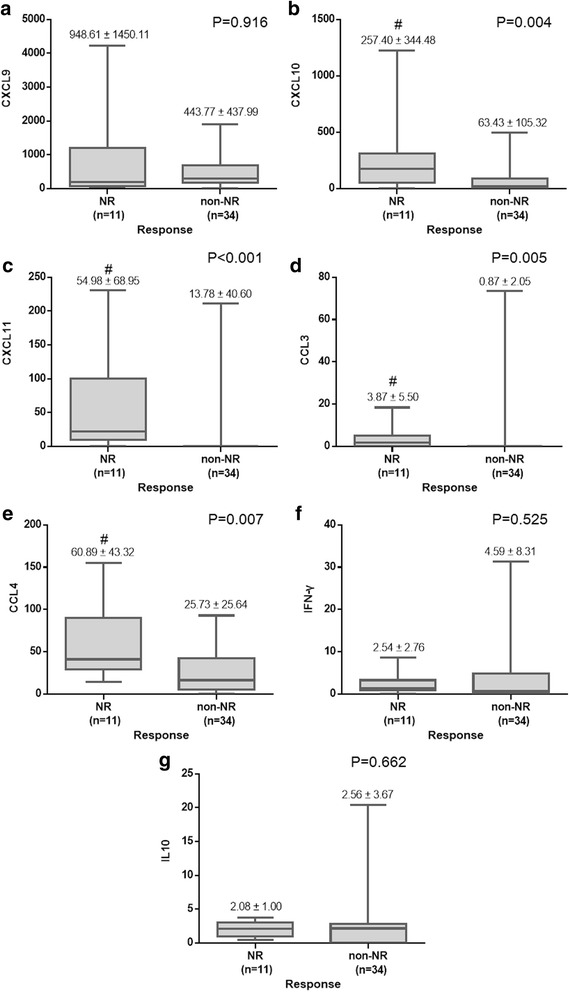
Comparison of Baseline Serum Levels of Chemokines/Cytokines of CHC, GT1 and IL28B-CC patients. **(a) (f) (g)** Pretreatment serum levels of CXCL9 (*P* = 0.916), IFN-γ (*P* = 0.525) and IL-10 (*P* = 0.662) showed no significant differences between NR and non-NR group of patients. **(b) (c) (d) (e)** NR group of patients showed higher baseline levels of CXCL10 (*P = 0.004), CXCL11 (*P < 0.001), CCL3 (*P = 0.005) and CCL4 (*P = 0.007) than non-NR group. Significance was assessed by means of the nonparametric Mann–Whitney test. Box plots represent medians and 25th–75th percentiles

The IL28B genotype polymorphism has significant impact on the treatment outcome with PegIFN/RBV but host immune factors for prediction of NR among the patients with advantageous rs12979860 CC allele were uncertained. Considering patients with rs12979860 CC allele, higher percentage of cirrhosis in the patients with NR was revealed (NR vs. non-NR = 45.5% vs. 17.6%, *p* = 0.039) (Table 3), and so were CXCL10, CXCL11, CCL3 and CCL 4 (CXCL10: NR vs. non-NR = 257.40 ± 344.48 vs. 63.43 ± 105.32, *p* = 0.004; CXCL11: NR vs. non-NR = 54.98 ± 68.95 vs. 13.78 ± 40.60, *p* < 0.001; CCL3: NR vs. non-NR = 3.87 ± 5.50 vs. 0.87 ± 2.05, *p* = 0.005; CCL4: NR vs. non-NR = 60.89 ± 43.32 vs. 25.73 ± 25.64, *p* = 0.007) (figure 2). CXCL10, CXCL11, CCL3, CCL4 and liver cirrhosis were the predictive factors for non-NR by univariate logistic analysis, but only the CCL4 was the independent predictor for non-NR by multivariate logistic analysis. (Table 4) Thus, our study indicated the advantageous genotype of IL28B is the only predictor for NR. As for patients with CC allele of rs12979860, higher baseline level of CCL4 is the only predictor for NR.

**Table 3:** Baseline Characteristics of CHC, GT1 and IL28B-CC patients

Variables	Overall	NR (11)^a^	non-NR (34)^a^	*P* value
Age (years)	59.60 ± 8.62	60.00 ± 7.01	59.47 ± 9.17	0.862
Male (%)	60.0	63.6	58.8	0.725
BMI (Kg/m^2^)	25.24 ± 3.01	25.56 ± 3.39	25.14 ± 2.92	0.693
AST (U/L)	75.76 ± 35.93	85 ± 42	73 ± 34	0.313
ALT (U/L)	98.82 ± 55.79	104 ± 56	97 ± 56	0.741
HCV RNA (log_10_ IU/ml)	2.39(6.22)^b^	4.09(12.27)^b^	3.97(5.56)^b^	0.927
Diabetes Mellitus (%)	26.7	13.6	32.3	0.072
Liver cirrhosis (%)	24.4	45.5	17.6	**0.039**

^a^number of patients shown in parentheses

^b^median (IQR) shown in parentheses

Data are shown as mean ± standard deviation. Statistic analysis was done by Mann-Whitney test for comparison. Significant *P* values are shown in bold. AST, aspartate aminotransferase; ALT, alanine aminotransferase; HCV, hepatitis C virus; IL28B, Interleukin-28B

**Table 4:** Predictors of NR in the patients of CHC GT1 and IL28B-CC, Treated with P/R by univariate and multivariate Logistic regression analysis

Variables	UV			MV		
	OR	95%C.I	*P* value	OR	95%C.I	*P* value
CXCL9	1.001	1.000–1.002	0.117			
CXCL10	1.006	1.000–1.012	**0.034**	0.985	0.965–1.005	0.150
CXCL11	1.014	1.000–1.029	**0.054**	1.020	0.995–1.046	0.118
CCL3	1.305	0.998–1.706	**0.052**	4.822	0.407–57.146	0.212
CCL4	1.032	1.007–1.057	**0.010**	1.134	1.006–1.277	**0.039**
IFN-γ	0.948	0.830–1.084	0.439			
IL10	0.943	0.720–1.236	0.673			
Liver cirrhosis	0.257	0.059–1.128	**0.072**	0.005	0.000–1.108	0.055

*UV* Univariate logistic regression analysis. *MV* Multivariate logistic regression analysis. *OR* Odds ratio, *CI* Confidence interval

Significant *P* values are shown in bold

## Discussion

In the present study, we focus on this group of patients with NR and found the rs12979860 non-CC genotype were strongly associated with treatment outcome of NR. Furthermore, in patients with advantageous rs12979860 CC genotype, higher baseline serum level of CCL4 was the only factor that is independently associated with non-response.

The role of IL28B genotype in predicting Peg/RBV treatment outcome like non-responder had been explored before like our previous studies [16, 17] and others [18–20]. Interesting, in the rapid advance of DAAs treatment, the role of IL28B on the SVR had gradually dwindled when treatment regimen are non- pegylated-IFN based [21]. However, in consideration of minor group with possible treatment failure by DAAs, the IL28B might still have impacts on the outcome [21].

The finding about chemokines be influential to the treatment outcome was compatible with another report that serum CXCL10 and CCL4 levels decreased significantly in GT-1 CHC patients with virological response [22]. Furthermore, CCL3, CCL4, CCL5, CXCL9, CXCL10 and CXCL11 were found to increase in both liver and peripheral blood during chronic hepatitis C in several studies [14, 22, 23]. The intra-hepatic levels of CXCL11 and CXCL10 were reported to correlate with HCV disease severity [13]. Patients with high CXCL10 at baseline were much less likely to achieve SVR, and the CXCL10 level was observed to be decreased following successful antiviral therapy [24, 25]. In HCV-infected livers, inflammation and fibrosis are mainly located in the portal areas, which may explain the up-regulation of CCL3–5 in the portal tracts [13]. However, the relationship existed between CCL3, CCL4 levels and the therapeutic responses were still controversial. A study showed that a low pretreatment CCL4 concentration was not only an independent predictor of early but also sustained virological response in CHC patients, while another study didn’t found significant differences [26, 27]. Interestingly, patients of advantageous IL28B genotype predominated among all recruited patients in the former study. To the best of our knowledge, no study yet had analyzed baseline CCL4 level in patient groups of advantageous IL28B genotype.

There were some limitations for this study. First of all, it was a retrospective study. However, in the new era of DAAs treatment, it is difficult to conduct a large-scale study just focused on PegIFN/RBV treatment. In addition, it is a medium-size study with case number of 60. However, in this scale of study, the serum levels of CCL4 become the only predictor for NR in patients with advantageous IL28B genotype. Therefore, it has emphasized the importance of CCL4 among other serum chemokines, especially in considering the future shorter duration of treatment for chronic hepatitis C patients receiving shorter duration of interferon-free DAAs.

## Conclusion

IL28B genotype is the predictor for non-responder in GT-1 CHC patients treated with PegIFN/RBV, while baseline serum level of CCL4 is the only predictor for non-responder in GT-1 CHC patients with favorable IL28B genotype.

## Abbreviations

CHC: Chronic hepatitis C
DAAs: Direct antiviral agents
EVR: Early virological responses
GT-1: Genotype-1
HCC: Hepatocellular carcinoma
IL28B: Interleukin-28B
NR: Non-response
PegIFN/RBV: Pegylated interferon plus ribavirin treatment
RVR: Rapid virological response
SVR: Sustained virological response
